# Effect of *COQ9* and *STAT5A* polymorphisms on reproductive performance in a Holstein cow herd in Mexico

**DOI:** 10.1590/1984-3143-AR2020-0039

**Published:** 2020-08-04

**Authors:** Néstor Gerardo Michel-Regalado, Miguel Ángel Ayala-Valdovinos, Jorge Galindo-García, Theodor Duifhuis-Rivera, David Román Sánchez-Chiprés, Mauricio Valencia-Posadas

**Affiliations:** 1 Departamento de Producción Animal, División de Ciencias Veterinarias, Centro Universitario de Ciencias Biológicas y Agropecuarias, Universidad de Guadalajara, Guadalajara, Jalisco, México; 2 Departamento de Veterinaria y Zootecnia, División de Ciencias de la Vida, Universidad de Guanajuato, Campus Irapuato Salamanca, Irapuato, Guanajuato, México

**Keywords:** Holstein, *COQ9* gene, *STAT5A* gene, fertility

## Abstract

Coenzyme Q9 (*COQ9*), a coenzyme Q (CoQ) precursor, is an essential component of the mitochondrial electron transport chain that drives adenosine triphosphate production. *COQ9* polymorphism 18:25527339 is characterized by substitution of guanine (allele G) for adenine (allele A), which modifies the function of the protein encoded by the gene. In Holsteins, allele A has been associated with better reproductive performance in terms of the conception rate, number of services per conception (SPC) and days open (DO). The signal transducer and activator of transcription (STAT) protein is a transcription factor activated in the presence of cytokines and growth factors. *STAT5A* polymorphism 19:42407732 in exon 8 has been associated with higher fertility and embryonic survival rates. The objective of this study was to determine the relationship of *COQ9* and *STAT5A* polymorphisms with reproductive parameters [calving to first heat interval (CFHI), DO and SPC]. Blood samples were taken from 112 lactating Holstein from a herd in México for allele genotyping by polymerase chain reaction-restriction fragment length polymorphism (PCR-RFLP). To estimate the association between reproductive parameters and genotypes, a linear mixed-effect model was performed. The *COQ9* AG genotype was associated significantly with lower SPC (P<0.05) but not with DO or CFHI. No significant association with any reproductive parameter was found for *STAT5A*. Our findings suggest that the *COQ9* 18:25527339 polymorphism is a useful molecular marker for improvement of reproductive performance in dairy herds.

## Introduction

Dairy cow selection and strong specialization for milk production have resulted in a significant reduction in the reproductive performance of dairy herds (Veerkamp et al., 2003). An understanding of the associations between polymorphic variants of the genes involved in reproduction and phenotypic features enables the use of selection strategies based on molecular markers, to improve animal productivity (Clempson et al., 2012). Some mutations affecting reproduction such as the single nucleotide polymorphism (SNP) allele A (18:25527339) in the coenzyme Q9 (*COQ9*) gene have been identified through association studies of the complete Holstein dairy cow genome (Ortega et al., 2016). The *COQ9* mutation is characterized by substitution of the nucleotide guanine for adenine, which changes the sequence of aspartic acid to asparagine in position 53 of the protein (Ortega et al., 2017). CoQ9 is known to binds with other molecules, such as CoQ7 to stabilize the CoQ synthesis complex (Lohman et al., 2014). CoQ is a critical component of the electron transport chain in the inner mitochondrial membrane; it transports electrons from complexes I and II to complex III (Turunen et al., 2004). Truncation or modification of the CoQ9 protein destabilizes the multiprotein complex and causes CoQ deficiency by reducing the activity of complexes I and III of the electron transport chain (Luna-Sánchez et al., 2015). CoQ9 has been found in tissues such as the endometrium, cumulus oophorus oocyte, and pre-implantation embryo. SNP 18:25527339 modifies cellular metabolic and energy efficiency, thereby modulating oocyte competence and embryo quality (Ortega et al., 2017).

The bovine gene *STAT5A* belongs to a family of placental lactogen and interferon-τ signal transducers and transcription activators. These molecules are specifically activated to regulate gene expression in the presence of cytokines and growth factors (Khatib et al., 2008). The interruption of STAT5 protein activity leads to corpus luteum deficiency and infertility. The corpus luteum produces the progesterone needed to support gestation (Teglund et al., 1998). *STAT5A* polymorphism 19:42407732 has been associated with reduced *in vitro* fertility rates, embryonic survival, and milk production and composition (Khatib et al., 2008). This embryonic death occurs at an early stage of development, and the molecular mechanism that controls it remains unknown. The objective of this study was to determine the relationships of the *COQ9* 18:25527339 and *STAT5A* 19:42407732 polymorphisms to reproductive parameters [calving to first heat interval (CFHI), days open (DO) and number of services per conception (SPC)] in lactating cows from a Holstein herd in Mexico.

## Methods

Laboratory analysis was done at the Institute of Animal Biotechnology of the University of Guadalajara, Mexico. The study was approved by Internal Bioethics Regulations of the University Center for Biological and Agricultural Sciences, University of Guadalajara, Mexico (Approval No. CC/NN11-12/00/2012).

We sampled 112 multiparous Holstein cows (26 in first lactation, 43 in second lactation, 22 in third lactation and 21 in fourth lactation) from a herd managed as part of an intensive production system in the municipality of San Juan de los Lagos, Jalisco, México. We obtained blood samples for DNA extraction from the caudal vein, collected in tube containing ethylenediaminetetraacetic acid as an anticoagulant (BD Vacutainer^®^ Systems, Plymouth, UK) and refrigerated at 4 °C until use. We kept the cows under similar management and feeding conditions, as recommended by the US National Research Council (NRC, 2001). The cows were milked three times a day with automated vacuum equipment. We selected only cows without partum dystocia or early postparturient diseases, such as placenta retention, clinical mastitis, metritis, clinical hypocalcemia and ketosis. We classified the cows by lactation number. We collected data on reproductive parameters (CFHI, DO and SPC) using the DairyCOMP 305 dairy management software (Steve Eicker & Connor Jameson, Tulare, CA, USA).

### DNA extraction and genotyping

We extracted genomic DNA from blood samples using the Quick-DNA™ Kit (Zymo Research, Orange CA, USA). For the genotyping of *COQ9* polymorphism 18:25527339 and *STAT5A* polymorphism 19:42407732, we used the primers and enzymes listed in Table 1. We performed polymerase chain reaction (PCR) as described by Ayala-Valdovinos et al. (2017). We amplified *COQ9* and *STAT5A* in a Techne^®^ TC-5000 thermal cycler (Techne Inc., Burlington, NJ, USA) using the following PCR protocol: initial denaturing at 95 °C for 5 min, followed by 35 cycles at 94 °C for 20 s, 54 °C (64 °C for *STAT5A*) for 30 s, 72 °C for 30 s and final extension at 72 °C for 5 min. We performed enzymatic digestion in the thermal cycler at 37 °C for 90 min. After digestion of the PCR products, we analyzed the fragments by agarose gel electrophoresis at 4% with GelRed^TM^ staining (Biotium, Hayward, CA, USA) and viewing under ultraviolet light.

**Table 1:** Primers used for genotyping.

**Gene**	**Primer**	**Sequence**	**Size (bp)**	**Enzyme**	**Reference**
*COQ9*	Forward	AGTTTCTGTTTCAGTGCCCCG	202	*Sau3AI*a	Own design
	Reverse	GCAGGTGTTCTGATGCCTACC			
*STAT5A*	Forward	GAGAAGTTGGCGGAGATTATC	820	*BstEII*a	Khatib et al. (2008)
	Reverse	CCGTGTGTCCTCATCACCTG			

bp: base pair.

^a^New England Biolabs, Inc., Ipswich, MA, USA.

### Statistical analysis

We assessed associations of *STAT5A* and *COQ9* genotypes with CFHI, DO, and SPC using the SAS 9.0 software (SAS Institute, Cary, NC, USA). We analyzed the data independently for each gene using a mixed model, with genotype and lactation number serving as fixed variables, and the animal serving as a random variable. We calculated differences between genotypes and lactations using the Tukey method (*P* < 0.05) as follows:

$$Y_{ijk}=µ+GENOTYPE_{i}+LACTATION_{j}+animal_{k}+\varepsilon_{ijk}$$ (1)

where Y_ijk_ is the observed value of the reproductive parameter (CFHI, DO, or SPC), µ is the general mean for the evaluated variable, GENOTYPE_i_ is the genotype for each gene of an animal (wild-type homozygote, heterozygote and mutant homozygote), LACTATION_j_ is lactation number for each animal (first, second, third, or fourth), animal_k_ is the random genetic component of each animal, and Ɛ_ijk_ is the experimental error. We performed a chi-Squared analyses of the gene and genotype frequencies using POPGENE software (version 1.32; UAlberta, 1997).

## Results

**Figure 1:**
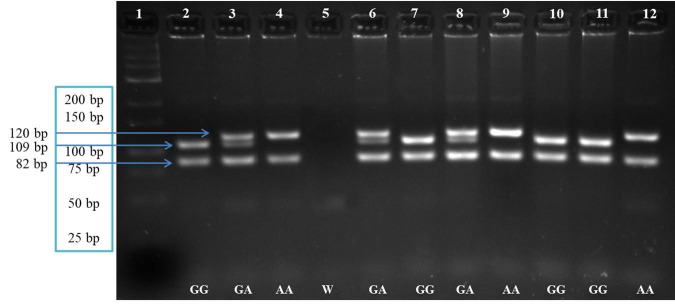
Agarose gel electrophoresis (4%) of *COQ9* gene fragments amplified by PCR-restriction fragment length polymorphism and digested with the *Sau3AI* enzyme. Lane 1 shows the molecular marker (25 bp, Thermo Fisher Scientific^®^). Lanes 2-4 are control samples for each genotype. Lane 5 shows reaction targets. Lanes 6-12 show cow samples analyzed in the study.

By PCR, we amplified a 202 bp *COQ9* DNA fragment. After enzymatic digestion, we identified three genotypes: AA (82 bp and 120 bp fragments), AG (82 bp, 109 bp, and 120 bp fragments), and GG (82 bp and 109 bp fragments; Figure 1). The frequencies of genotypes AA, AG, and GG in the study population were 0.18, 0.56, and 0.26, respectively. The genic frequency was slightly higher for allele G (0.54) than for allele A (0.46).

**Figure 2:**
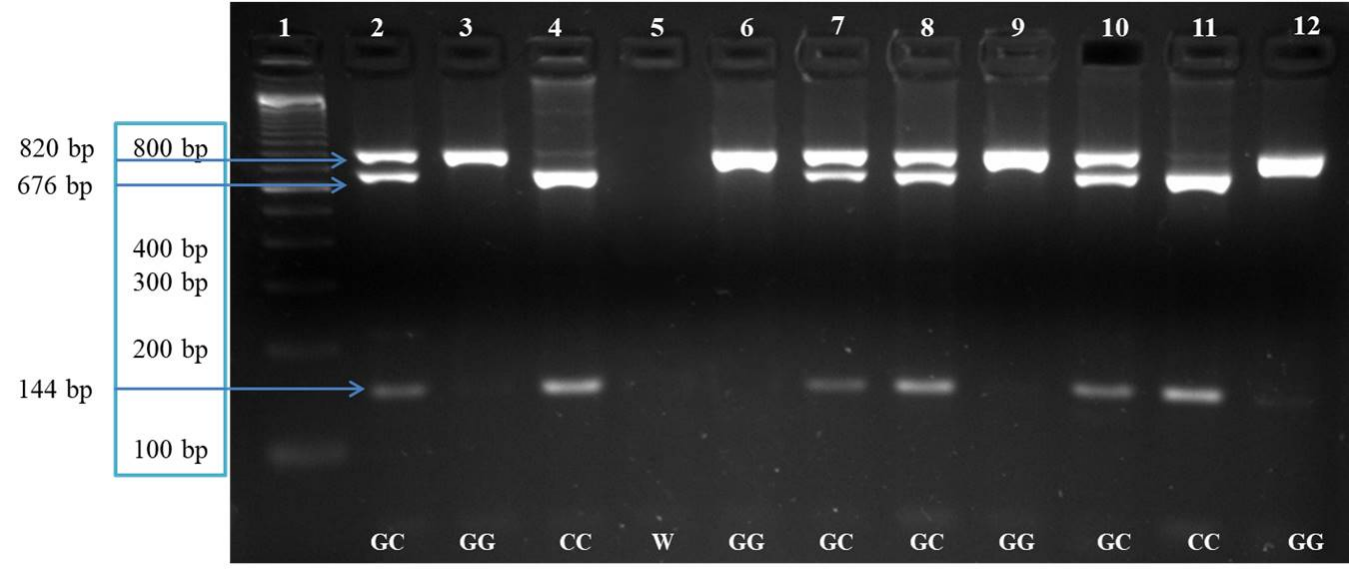
Agarose gel electrophoresis (3%) of *STAT5A* gene fragments amplified by PCR-restriction fragment length polymorphism and digested with the *Eco91I* enzyme. Lane 1 shows the molecular marker (100 bp, Thermo Fisher Scientific^®^). Lanes 2-4 are control samples for each genotype. Lane 5 shows reaction targets. Lanes 6-12 show cow samples analyzed in the study.

For *STAT5A*, we identified three genotypes: CC (676 bp and 144 bp fragments), GC (820 bp, 676 bp, and 144 bp fragments), and GG (820 bp fragment; Figure 2) the frequencies of the CC, GC, and GG genotypes were 0.33, 0.51, and 0.16, respectively. The allele with the highest frequency was wild-type allele C (0.58). The frequency of the mutant allele G was 0.42. Observed frequencies for both polymorphisms were in Hardy-Weinberg equilibrium.

Genotypes were associated significantly with the lactation number and CFHI (both *P* < 0.05). The confidence interval for CFHI was smallest for the fourth lactation group (Table 2). The heterozygous *COQ9* genotype was associated significantly with SPC (*P* < 0.05); heterozygous cows needed the fewest inseminations per conception. For the *STAT5A* polymorphism, we found no significant association between any genotype and reproductive parameter analyzed.

**Table 2:** Least square mean CFHI, DO, and SPC values by genotype and lactation number.

**CFHI**	***COQ9* Genotype**	**(days)±EE**		***STAT5A* Genotype**	**(days)±EE**		**Lactation number**	**(days)±EE**	
	AA	72.9	5.1^a^	CC	66.5	4.6^a^	L1	73.9	7.1^a^
	AG	69.2	4.2^a^	CG	69	4^a^	L2	71.3	5.3^a^
	GG	69.2	4.6^a^	GG	73.6	5.4^a^	L3	75.8	3.9^a^
							L4	60.9*	3.8^b^
**DO**	***COQ9* Genotype**	**(days)±EE**		***STAT5A* Genotype**	**(days)±EE**		**Lactation number**	**(days)±EE**	
	AA	127.6	12.7^a^	CC	111.1	9.3^a^	L1	126	11.1^a^
	AG	103.5	7.5^b^	CG	108.3	7.5^a^	L2	116	8.7^a^
	GG	128.7	9.7^a^	GG	124	13.2^a^	L3	122.2	12.3^a^
							L4	115.3	12.4^a^
**SPC**	***COQ9* Genotype**	**±EE**		***STAT5A* Genotype**	**±EE**		**Lactation number**	**±EE**	
	AA	3.6	0.5^a^	CC	3.1	0.4^a^	L1	3.4	0.4^a^
	AG	2.5*	0.3^b^	CG	2.8	0.3^a^	L2	3.1	0.3^a^
	GG	3.7	0.4^a^	GG	3.3	0.5^a^	L3	3.1	0.5^a^
							L4	3.4	0.5^a^

Different letter within columns indicate significant differences (Tukey method, *P* < 0.05).

* *P* < 0.05. CFHI: calving to first heat interval; EE: mean standard error; DO: days open; SPC: number of services per conception.

## Discussion

We studied the association of *COQ9* and *STAT5A* polymorphisms with three reproductive parameters: CFHI, DO, and SPC. For the *COQ9* polymorphism, the frequencies of alleles A and G were 0.46 and 0.54, respectively. Ortega et al. (2016) reported similar frequencies of 0.49 for allele A and 0.51 for allele G. For the *STAT5A* polymorphism, the frequencies of allele C and G were 0.58 and 0.42, respectively. Shirasuna et al. (2011) reported frequencies of 0.56 for allele C and 0.44 for allele G and Hax et al. (2017) reported frequencies of 0.52 and 0.48, respectively.

We also found that cows with the *COQ9* genotype AG had the lowest SPC, and that genotypes AA and GG were not associated with any parameter analyzed. In a human chorionic gonadotropin stimulation study, Zolini et al. (2019) found that cows with *COQ9* genotype AG had the highest pregnancy rates at 30 and 60 days after artificial insemination, whereas those with genotype AA had the lowest pregnancy rate. Ortega et al. (2017) found that cows with *COQ9* genotype AA had lower SPC, and DO values, a higher pregnancy rate and more efficient mitochondrial function. These cows produced higher concentrations of adenosine triphosphate, which intensifies the cellular response to hormone stimulation, triggering the release of prostaglandins and subsequent lysis of the corpus luteum (Zolini et al., 2019). The differences in genotype behavior may be due to particular effects of dominance or heterosis in each population (Khayatzadeh et al., 2018), whereas the influence of allele A is constant and independent of genotype. One explanation for the observed reproductive advantage of heterozygous cows in our study is that the alleles may be favorable for different follicular growth phases, resulting in the maximization of reproductive potential (Ortega et al., 2017).

We found no significant association between *STAT5A* polymorphism 19:42407732 and CFHI, DO, or SPC. Khatib et al. (2008) reported significant associations of this polymorphism with the *in vitro* pregnancy and embryonic survival rates. The lack of reproductive parameter effect of this polymorphism in our study may be due to our use of different populations and parameters than did Khatib et al. (2008), as well as different fertilization methods and sample sizes, which might change the reproductive performance of the animals (Maillo et al., 2016). As in our study, Oikonomou et al. (2011) found no association of SPC or DO with any *STAT5A* genotype. The similarity of these results may reflect the use of rectal palpation to diagnose pregnancy at 45 days after artificial insemination in both studies. This approach may have resulted in the discounting of embryonic mortality which occurs at a high rate prior to 45 gestational days (Diskin et al., 2016), thereby masking the effect of the polymorphism. Conversely, Shirasuna et al. (2011) and Hax et al. (2017) found lesser CFHIs in cows with allele G of the *STAT5A* polymorphism. The effect of this allele may be due to the gene’s role in the growth hormone signaling pathway, as STAT proteins are activated specifically to regulate gene transcription, increasing the insulin-like growth factor 1 level. This hormone increases estradiol production in the follicle, thereby triggering early ovulation (Butler et al., 2004). Homer et al. (2013) found that cows with STAT5A allele G showed greater in-stall estrus expression, which can also be due to increased production of estradiol in the follicle. Greater estrus expression is associated with greater fertility and improved CFHI, DO, and SPC (Burnett et al., 2018). We propose that future studies include larger numbers of animals and involve the measurement of hormones involved in the growth hormone metabolic pathway to determine the full effect of *STAT5A* on reproductive performance.

In this study, CFHIs were lesser in fourth-lactation cows. In general, multiparous cows show better reproductive performance. Tanaka et al. (2008) reported lesser CFHIs in multiparous than in primiparous cows, which have nutritional requirements for growth as well as lactation. According to Adrien et al. (2012) the body conditions of multiparous cows in the days preceding labor are regulated more easily, which contributes to CFHI reduction. In addition, incidences of dystocia and reproductive diseases are lower in multiparous than in primiparous cows (Gröhn and Rajala-Schultz, 2000).

## Conclusion

In conclusion, *STAT5A* polymorphism 19:42407732 was not associated with the reproductive parameters evaluated in this study of a Holstein cow herd in Mexico. Genotype AG of *COQ9* polymorphism 18:25527339 was associated with lower SPC and is suggested as a molecular marker to improve Holstein cow reproductive performance in dairy herds in Mexico.
